# Synchrony of plant cellular circadian clocks with heterogeneous properties under light/dark cycles

**DOI:** 10.1038/s41598-017-00454-8

**Published:** 2017-03-22

**Authors:** Masaaki Okada, Tomoaki Muranaka, Shogo Ito, Tokitaka Oyama

**Affiliations:** Department of Botany, Graduate School of Science, Kyoto University, Kitashirakawa-oiwake-cho, Sakyo-ku, Kyoto 606-8502 Japan

## Abstract

Individual cells in a plant can work independently as circadian clocks, and their properties are the basis of various circadian phenomena. The behaviour of individual cellular clocks in *Lemna gibba* was orderly under 24-h light/dark cycles despite their heterogeneous free-running periods (FRPs). Here, we reveal the entrainment habits of heterogeneous cellular clocks using non-24-h light/dark cycles (T-cycles). The cellular rhythms of *AtCCA1::LUC* under T = 16 h cycles showed heterogeneous entrainment that was associated with their heterogeneous FRPs. Under T = 12 h cycles, most cells showed rhythms having ~24-h periods. This suggested that the lower limit of entrainment to the light/dark cycles of heterogeneous cellular circadian clocks is set to a period longer than 12 h, which enables them to be synchronous under ~24-h daily cycles without being perturbed by short light/dark cycles. The entrainment habits of individual cellular clocks are likely to be the basis of the circadian behaviour of plant under the natural day–night cycle with noisy environmental fluctuations. We further suggest that modifications of *EARLY FLOWERING3* (*ELF3*) in individual cells deviate the entrainability to shorter T-cycles possibly by altering both the FRPs and light responsiveness.

## Introduction

The circadian clock maintains biological rhythms with a period of approximately 24 h, even without external cues, such as under continuous dark conditions (DD). The period length of the circadian rhythms measured under constant conditions is called the free-running period (FRP) and varies among organisms, tissues, or even cells of the same cell type^1, 2^. However, circadian rhythms can be entrained to the external daily cycle. The entrainment ranges of various organisms’ circadian rhythms have been described and plants showed a range between 15 and 35 h^3^. This entrainability is a critical property of the circadian clock that allows it to adjust to the environmental cycle.

In plants, the circadian rhythm can be determined through the oscillation of clock-related genes, which form transcription–translation feedback loops coupled with the light signalling of photoreceptors^4^. Thus, each cell in a plant body can be regarded as a cell-autonomous circadian clock. We have established a single-cell bioluminescence monitoring system for circadian rhythms using monocotyledonous duckweed and reported that individual cells harbouring *AtCCA1::LUC* had circadian rhythms with a relatively wide range of FRPs, even in the same tissue^2, 5^. Individual cells are capable of independently resetting their own rhythms to a light signal^2^. Heterogeneous FRPs were masked under light/dark cycles with a period of 24 h. This phenomenon seems to be the basis of the entrainment of circadian rhythms at the whole plant level. Its mechanisms, which include photoreceptors and clock-related genes as components, have been genetically analysed using *Arabidopsis* ^6^. EARLY FLOWERING3 (ELF3) of *Arabidopsis* modulates phytochrome signalling and is a key factor for the entrainment of circadian rhythms to light/dark cycles^7^. This nuclear protein also works as a component of the Evening Complex in transcription–translation feedback loops^8–10^. It is essential for circadian rhythmicity, and the null *elf3-1* mutant has an arrhythmic phenotype^11^. Moreover, a reduced-function allele, *elf3-12*, shows a shorter period length and *ELF3* overexpression lengthened the period under continuous light conditions (LL), suggesting that *ELF3* controls the periodicity of the circadian rhythm in *Arabidopsis* ^7, 9^. The null *elf3-1* mutant also has a phenotype of acute circadian responses to light signals^7^. Thus, *ELF3* is involved in time-dependent restrictions (circadian gating) of the light sensitivity critical to the entrainability of the clock system to light/dark cycles^12^. These functions of *ELF3* appear to be conserved in higher plants, because the genetic modification of *LgELF3* in the duckweed plant, *Lemna gibba*, results in circadian phenotypes related to those of *Arabidopsis* ^13, 14^.

Here, we unveil the entrainment habits of plant cellular clocks using a non-24-h light/dark cycle treatment. We also show the critical involvement of *ELF3* in the entrainment by modulating the FRP and light responsiveness.

## Results

### Evaluation of *ELF3* functions in the maintenance of circadian rhythmicity and the regulation of its FRP at the single-cell level

**Figure 1:**
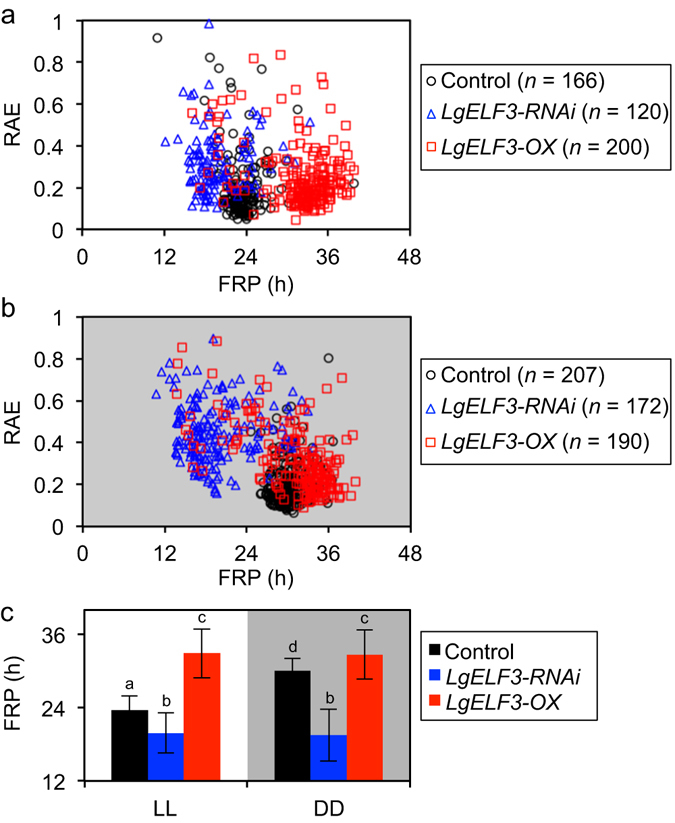
Changes in FRPs and RAEs of cellular *AtCCA1::LUC* rhythms caused by *LgELF3* effectors under constant conditions. FRPs and RAEs of individual cellular rhythms under LL (**a**) and DD (**b**) are plotted for the control vector (*pBI221ΔGUS*, black circles), the knockdown *LgELF3-RNAi* construct (blue triangles) and the overexpressing *LgELF3-OX* construct (red squares). Three independent experiments for each condition were conducted, and the total samples were plotted. (**c**) Comparison of FRPs shown in (**a**) and (**b**) for bioluminescence traces with RAE values less than 0.4. Data are expressed as mean ± SD values. Means with the same letters are not significantly different. *p < 0.01, significant differences between the mean values are based on a two-level nested ANOVA model with six groups [effector constructs (control, *LgELF3-RNAi*, *LgELF3-OX*) under LL or DD], and subgroups (individual plants in the three experiments) nested within the groups.

We first evaluated the effects of the knockdown and overexpression of *LgELF3* on the FRP of the *AtCCA1::LUC* rhythm in individual cells of *L. gibba* plants. We conducted single-cell bioluminescence monitoring and functional analyses by co-transfecting an effector, *LgELF3H1-RNAi* (knockdown; *LgELF3-RNAi*) or *LgELF3H1-ox* (overexpresser; *LgELF3-OX*), with the reporter^13^. Under LL, the bioluminescent reporter that was co-transfected with the control vector (*pBI221ΔGUS*) showed robust circadian rhythms for individual cells, with the peaks at subjective dawn. Their average bioluminescence indicated a weakly damped rhythm (Supplementary Fig. 1). We analysed each cellular circadian rhythm using the fast Fourier transform non-linear least squares (FFT-NLLS) method and calculated the FRP and the relative amplitude error (RAE) (Fig. 1a). The RAE value represents the degree of confidence, ranging from 0 (complete sine-fitting) to 1 (arrhythmic)^15^. More than 90% of luminescent cells showed robust bioluminescence rhythms with RAE values of less than 0.4 under LL (Fig. 1a; Table 1). The mean FRP of those cellular rhythms was estimated as 23.6 h (Table 1).

**Table 1:** Summary of the circadian properties of individual cells in co-transfection assays with the *AtCCA1::LUC* reporter.

Effector	Under continuous light conditions (LL)				Under continuous dark conditions (DD)			
	No. of cells	% of RAE < 0.4	Mean FRP (h)*	1^st^ peak time (h)**	No. of cells	% of RAE < 0.4	Mean FRP (h)*	1^st^ peak time (h)**
Control (*pBI221ΔGUS*)	166	92.2	23.6 ± 2.3	13.4 ± 1.7	207	93.7	30.1 ± 2.0	17.0 ± 2.1
*LgELF3-RNAi*	120	77.5	19.8 ± 3.3	13.7 ± 3.0	172	40.1	19.5 ± 4.2	11.0 ± 3.2
*LgELF3-OX*	200	87.5	32.9 ± 4.0	15.7 ± 3.7	190	74.7	32.7 ± 4.1	17.4 ± 5.4
Control (*pUC18-Cas9*)	142	95.8	24.6 ± 2.7	15.9 ± 2.1	not tested	not tested	not tested	not tested
sg*LgELF3*	110	73.6	20.8 ± 3.1	15.1 ± 1.9	not tested	not tested	not tested	not tested

*Means ± S.D. were calculated using data of cells with relative amplitude error (RAE) values < 0.4.

**Means ± S.D. were calculated using data of peak times in the range between 0 h and 24 h. Time 0 h was the onsets of measurement shown in Supplementary Figs 1 and 2.

The co-transfection of *LgELF3-RNAi* severely affected the bioluminescence rhythms of individual cells and the average of their bioluminescence traces under LL (Fig. 1a; Supplementary Fig. 1; Table 1). The RAE values of those traces were relatively high when compared with those of the control: 77.5% of luminescent cells showed rhythms with RAE values of less than 0.4 (Fig. 1a; Table 1). Their bioluminescence traces appeared disordered. Even low-RAE cells showed unstable rhythms with shorter period lengths and higher trough levels (Supplementary Fig. 1). The mean FRP of individual cellular rhythms was estimated as 19.8 h (Table 1). This indicated that the knockdown of *LgELF3* by the RNAi construct resulted in shorter period lengths. This phenomenon is consistent with the shorter period phenotype of the weak *elf3* allele in *Arabidopsis* ^9^. FRPs of individual cells varied and the SD value (σ = 3.3 h) was larger than that of the control (σ = 2.3 h), suggesting that the FRP was destabilized in *LgELF3*-*RNAi*-transfected cells. The damping phenomenon shown in the circadian rhythm of the average of individual bioluminescence traces appeared to be due to this instability in the period lengths of individual cells. Notably, despite the deficient circadian properties in *LgELF3*-*RNAi*-transfected cells under LL, the first peak times near the end of the 12-h dark period were similar to those of the control (Table 1). Thus, the circadian rhythms of many *LgELF3*-*RNAi*-transfected cells appeared to be normally reset by the light/dark cycle. We also applied a CRISPR/Cas9 system to the cellular bioluminescence monitoring system as an alternative method to disrupt the *LgELF3* gene^16^. The target sequence of sg*LgELF3* was designed in the conserved N-terminal region of *ELF3*. Co-transfection of the CRISPR/Cas9 system severely affected cellular circadian rhythms (Supplementary Fig. 2). The rates in rhythmic cells and distributions of FRPs in sg*LgELF3*-transfected cells were comparable to those of *LgELF3-RNAi*-transfected cells, suggesting that the effects of *LgELF3-RNAi* on the bioluminescence rhythms were as severe as those of the CRISPR/Cas9 system targeting *LgELF3* (Fig. 1a; Supplementary Fig. 2; Table 1).

The average bioluminescence of *LgELF3-OX*-transfected cells under LL showed a damped rhythm with a longer period length compared with the control (Supplementary Fig. 1). Approximately 90% of luminescent cells showed robust rhythms with RAE values of less than 0.4 (Fig. 1a; Table 1). The average of their FRPs was estimated as 32.9 h with a larger SD value (σ = 4.0 h) than that of the control (σ = 2.3 h, Fig. 1c; Table 1). The first peaks were delayed by ~2 h when compared with those of the control, and their SD value (σ = 3.7 h) was larger than that of the control (σ = 1.7 h, Table 1). These results suggested that *LgELF3* overexpression lengthened the FRP of cellular rhythms and impaired the light-dependent resetting system. The dramatic changes in FRPs of cellular rhythms observed both in *LgELF3-RNAi-* and *LgELF3-OX*-transfected cells suggested that *LgELF3* played an important role in the modulation of FRPs under LL.

Under DD, the bioluminescent reporter in the control showed damped cellular circadian rhythms with FRPs averaging 30.1 h (Fig. 1b; Supplementary Fig. 1; Table 1), or 6.5 h longer than under LL (Fig. 1c; Table 1). Meanwhile, such light-dependent differences in the period lengths disappeared both in *LgELF3-RNAi-* and *LgELF3-OX*-transfected cells (Fig. 1; Supplementary Fig. 1; Table 1). The FRPs of *LgELF3-RNAi-* and *LgELF3-OX*-transfected cells were relatively close to those of the control under LL and DD, respectively (Fig. 1c; Table 1). These suggested that the *LgELF3* was inactive under LL and active under DD in the light-dependent modulation of the FRP. The first peaks for *LgELF3-RNAi*-transfected cells under DD were ~6 h earlier compared with the control. The difference in their peak phases seemed to result from the differences in their period lengths under DD. For *LgELF3-OX-*transfected cells, the average of the first peak times was similar to that of the control under DD, however their SD value (σ = 5.4 h) was larger than that of the control (σ = 2.1 h, Table 1). This much larger SD value suggested that *ELF3* overexpression impaired the resetting of cellular circadian rhythms by the light/dark signal.

### Entrainability of the circadian rhythms to short photoperiods at a whole plant level

**Figure 2:**
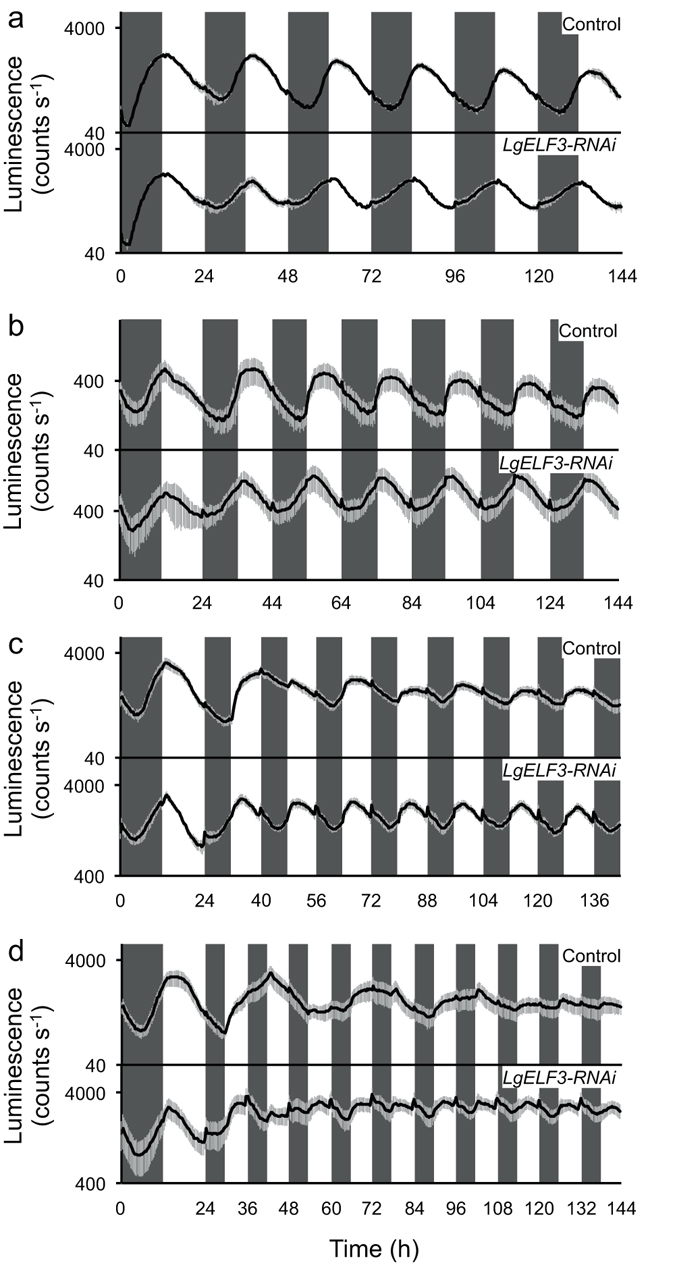
Entrainability of the circadian rhythm to various T-cycles at a whole plant level and its alteration by the *LgELF3* knockdown. The bioluminescence of the *AtCCA1::LUC* reporter was measured under various light/dark cycles (T-cycles), T = 24 h (**a**), 20 h (**b**), 16 h (**c**) and 12 h (**d**). Black lines and grey bars represent the mean value and SD values, respectively. Three independent experiments with three or four replicate dishes each were conducted and a representative trace for each condition is shown. The shaded boxes indicate the duration of the dark periods in the light/dark cycles.

Circadian rhythms are generally entrained to light/dark environmental cycles of a range of period lengths including 24 h. To demonstrate the entrainability of circadian rhythms of *L. gibba*, we performed T-cycle–non-24 h light/dark cycle experiments^17^. First, the *AtCCA1::LUC* rhythms of the control samples were monitored at the whole plant level under the following T-cycle conditions: T = 24 h, 20 h, 16 h and 12 h (upper panels in Fig. 2a–d). Under T = 24 h and 20 h cycles, the bioluminescence rhythms were clearly entrained to the light/dark cycles with peaks at dawn, (upper panels in Fig. 2a and b). The trough phases of these rhythms were ~18 h (T = 24 h), or ~17 h (T = 20 h) after light onset, and the timing was almost the same in every cycle at each condition (Supplementary Fig. 3). The autocorrelation functions of these bioluminescence traces showed the highest peaks at the time lag corresponding to the periods of the T-cycles (Supplementary Fig. 3). Under T = 16 h cycles, the bioluminescence showed a complex behaviour (upper panel in Fig. 2c). During the second T-cycle (Time 40–56 h in Fig. 2c), the bioluminescence retained a high intensity with a moderate decrease and no clear rhythmicity. After the cycle, the bioluminescence showed a low amplitude rhythm compared with in T = 24 h and 20 h cycles. The trough phases of the rhythms fluctuated every cycle at around 14 h after light onset, suggesting that the bioluminescence rhythm was not fully entrained to the T = 16 h cycles (Supplementary Fig. 3). Under T = 12 h cycles, the bioluminescence traces of the *AtCCA1::LUC* showed a damped rhythm with a period length longer than 24 h (upper panel in Fig. 2d). Their trough timing was not fixed to a phase in the light/dark cycles and their autocorrelation functions showed peaks at time lags of 24 h or longer, without apparent peaks at around 12 h (Supplementary Fig. 3). Thus, the *AtCCA1::LUC* rhythm failed to be entrained to the T = 12 h cycles, and this short T-cycle appeared to be ignored.

Next, we examined the effects of the *LgELF3* knockdown on the entrainability of the circadian rhythm at the whole plant level under T-cycle conditions. The *ELF3* gene is deeply involved in the determination of the FRP and the light resetting of the circadian clock system (Fig. 1; Table 1)^7, 9^, and the entrainability is dependent on these characteristics^6, 17, 18^. Under T = 24 h, 20 h and 16 h, the *AtCCA1::LUC* reporter, co-transfected with the *LgELF3-RNAi* construct, showed rhythms that were clearly entrained to the light/dark cycles (lower panels in Fig. 2a–c). The autocorrelation functions of these bioluminescence traces showed that the highest peaks were at the time lag corresponding to the period of the T-cycles (Supplementary Fig. 3). The trough phases of the rhythms were almost fixed at ~11 h, ~12 h and ~13 h after light onset for T = 24 h, 20 h and 16 h cycles, respectively. Under T = 24 h and 20 h cycles, the *LgELF3* knockdown resulted in phase advances compared with the control (Supplementary Fig. 3). In the *LgELF3*-knockdown cells, this phase advance might be associated with the short FRP. Under T = 16 h cycles, unlike the labile and low-amplitude bioluminescence rhythms of the control, the rhythms of the *LgELF3* knockdown were robust constantly from the first T = 16 h cycle (Fig. 2c). Under T = 12 h cycles, the bioluminescence trace of the *LgELF3* knockdown showed a low amplitude rhythm with a period length of ~12 h (Fig. 2d; Supplementary Fig. 3). In addition to transient light and dark responses, the bioluminescence decreased during the 6-h dark period of each light/dark cycle. The trough timing was near light onset but varied cycle by cycle (Fig. 2d; Supplementary Fig. 3). The autocorrelation functions were totally different between the *LgELF3* knockdown and the control. The *LgELF3* knockdown showed a period component of 12 h, although no positive autocorrelations were found in the control samples at around the 12 h time lag. Thus, unlike the control, the *LgELF3* knockdown appeared to be partially entrained to this short T-cycle (Supplementary Fig. 3).

### Entrainability of cellular circadian clocks to shorter photoperiods

**Figure 3:**
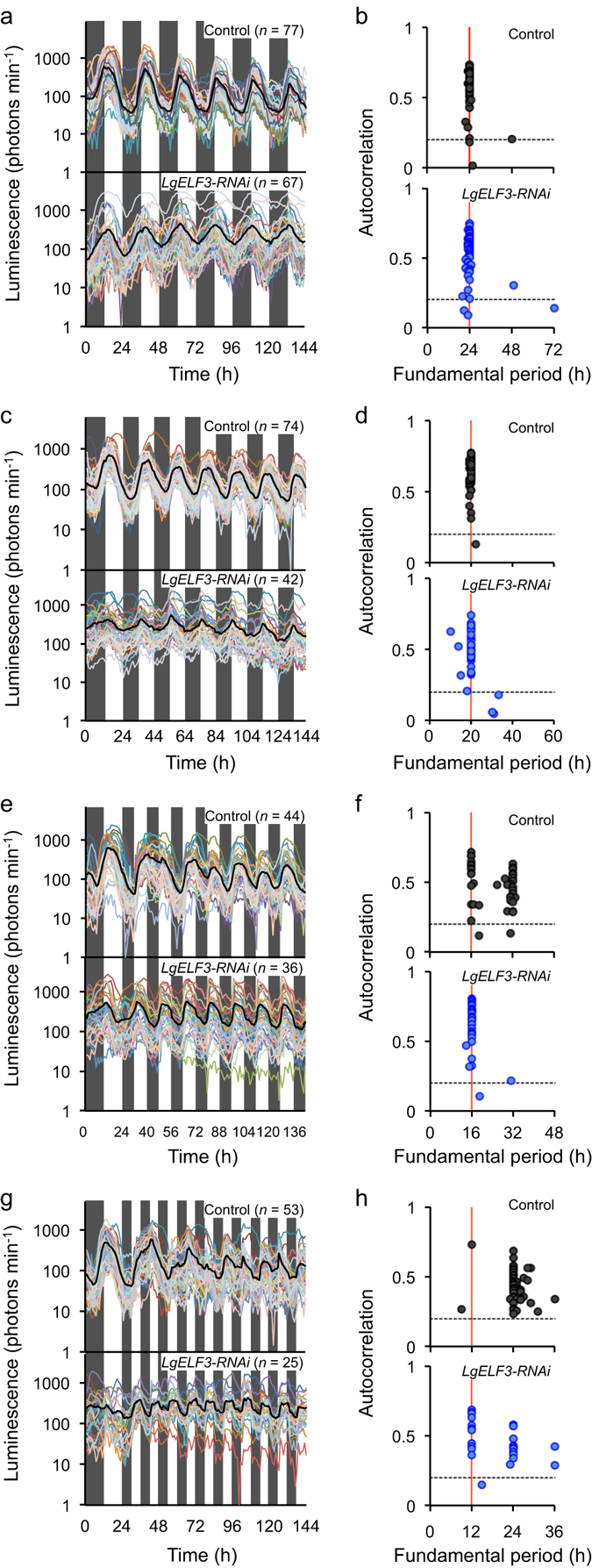
Entrainability of the circadian rhythm to T-cycles in individual cells and its alteration by the *LgELF3* knockdown. (**a,c,e,g**) Bioluminescence traces of the *AtCCA1::LUC* reporter obtained by the single-cell bioluminescence monitoring under various T-cycles, T = 24 h (**a**), 20 h (**c**), 16 h (**e**) and 12 h (**g**), for every measured cell on a frond. A black line in each graph represents the average bioluminescence of the total traces. Shaded boxes indicate the duration of the dark periods in the light-dark cycles. Three experiments were conducted for each condition, except for the T = 24 h condition that had data from two experiments, and a representative trace for each condition is shown. (**b,d,f,h**) Comparison of the fundamental periods of individual bioluminescence traces in T-cycle conditions. The highest peak value of the autocorrelation function of each bioluminescence trace (from 48 h to 144 h) in the graph on the left is plotted against the time lag (fundamental period). The horizontal dashed black lines and the vertical red lines in each graph represent the borders of the 95% confidence intervals of the autocorrelation function and the period lengths of the T-cycles, respectively.

Individual cellular circadian rhythms, even in the same tissue, showed heterogeneity in their circadian properties under constant conditions (Fig. 1)^2^, and we assumed that individual cells in a plant body might show rhythms differently entrained in T-cycle conditions. First, the *AtCCA1::LUC* bioluminescence of individual cells was monitored under the following T-cycle conditions: T = 24 h, 20 h, 16 h and 12 h (upper panels in Fig. 3a,c,e and g). The autocorrelation function of each bioluminescence trace was calculated (Supplementary Figs 4, 5 and 6). The time lag with the highest peak in the autocorrelation function was regarded as the “fundamental period” of the bioluminescence trace. The autocorrelation value at the time lag was plotted against the fundamental period for every measured cell (upper panels in Fig. 3b,d,f and h). Under T = 24 h and 20 h cycles, almost all of the luminescent cells showed rhythms that were clearly entrained to the light/dark cycles, and the fundamental periods of those rhythms were estimated as values corresponding to the T-cycle’s period (Supplementary Figs 4 and 7; upper panels in Fig. 3a–d).

Under T = 16 h cycles, bioluminescence traces varied between cells. Most cells showed a peak in every 8-h light period. However, in a portion of the cells (67/177 cells or 38%), the luminescence intensities at peaks regularly fluctuated, having high and low values (Supplementary Figs 5 and 7; upper panel in Fig. 3e). This suggested that the cells with the fluctuating peak intensities were unlikely to be entrained to the T = 16 h cycles, and the states of their cellular clock alternated every other cycle. Fundamental periods of cellular bioluminescence traces in a frond were roughly classified into two groups of ~16 h and ~32 h (the 16-h and 32-h groups, respectively), suggesting that the entrainability of the circadian rhythm varied among individual cells, even in the same plant (upper panel in Fig. 3f). Cells in both groups were widespread on the frond, suggesting that the variation of the entrainability of cellular rhythms did not depend on their physical positions (Supplementary Fig. 8), but appeared to result from heterogeneous circadian properties among individual cells.

When plants were transferred from 12-h dark/12-h light to 6-h dark/6-h light (T = 12 h cycles) conditions, the bioluminescence of most cells reached its first peak during the second light period (Supplementary Fig. 6; Time 42–48 h in the upper panel in Fig. 3g). Thus, those plants likely did not regard the first 6-h dark/6-h light cycle as a day–night cycle. In the third and later cycles, the bioluminescence traces of many cells had rhythms that only peaked every 24 h or longer. Their autocorrelation functions showed peaks at a time lag of 24 h (38/159 cells or 24%) or longer (61/159 cells or 38%), without a peak at 12 h (Supplementary Figs 6 and 7). Cellular rhythms with a fundamental period of 24 h (=2 T) looked to be entrained to every second cycle (known as frequency demultiplication)^17^; those with longer fundamental periods were not apparently entrained to the T-cycle and seemingly freeran. This result was consistent with the T = 12 h cycles being ignored at the whole plant level (Fig. 2d). Meanwhile, some cells peaked during the 6-h light period of each light/dark cycle after the third cycle, and their luminescence intensities fluctuated (Supplementary Fig. 6). Their autocorrelation functions showed bimodalities, with peaks at time lags of 12 h and 24 h; fundamental periods of most of them were 24 h (Supplementary Figs 6 and 7; upper panel in Fig. 3h). Thus, it was unlikely that those cellular circadian rhythms were entrained to the T = 12 h cycles.

We next examined the effects of knocking down *LgELF3* on the *AtCCA1::LUC* rhythm of individual cells under T-cycle conditions. Under T = 24 h and 20 h cycles, *LgELF3-RNAi*-transfected cells showed rhythms that were clearly entrained to the light/dark cycles, having advanced troughs compared with those of the control (Fig. 3a and c; Supplementary Figs 4 and 7). The fundamental periods of those cellular rhythms corresponded with the T-cycles (lower panels in Fig. 3b and d). Under T = 16 h cycles, bioluminescence traces of *LgELF3-RNAi*-transfected cells looked similar, and the fundamental periods of most cells were 16 h (Supplementary Figs 5 and 7; lower panels in Fig. 3e and f). This period uniformity was in contrast to the heterogeneous fundamental periods, 16-h and 32-h, of control cells under the same conditions (Fig. 3f). The entrainability of *LgELF3*-knockdown cells to T = 16 h cycles appeared to be consistent with the shorter FRPs of those cells (Fig. 1). When plants were transferred from 12-h dark/12-h light to 6-h dark/6-h light (T = 12 h cycles) conditions, the bioluminescence of most *LgELF3-RNAi*-transfected cells reached its first peak during the first light period (Supplementary Fig. 6; Time 30–36 h in the lower panel in Fig. 3g). In contrast, the control cells did not show obvious peaks during this light period (Supplementary Fig. 6). The bioluminescence traces of the *LgELF3-RNAi*-transfected cells showed a peak during the 6-h light period of every light/dark cycle throughout the monitoring. Most of them showed a peak at the time lag of 12 h with autocorrelation functions though fundamental periods of half of them were estimated at 24 h (Supplementary Figs 6 and 7). Thus, the knockdown of *LgELF3* impaired the proper entrainability that enables cellular circadian clocks to maintain their period lengths close to 24 h, without being perturbed by short light/dark fluctuations.

### Heterogeneity in the entrainment of cellular circadian clocks is associated with their FRPs

**Figure 4:**
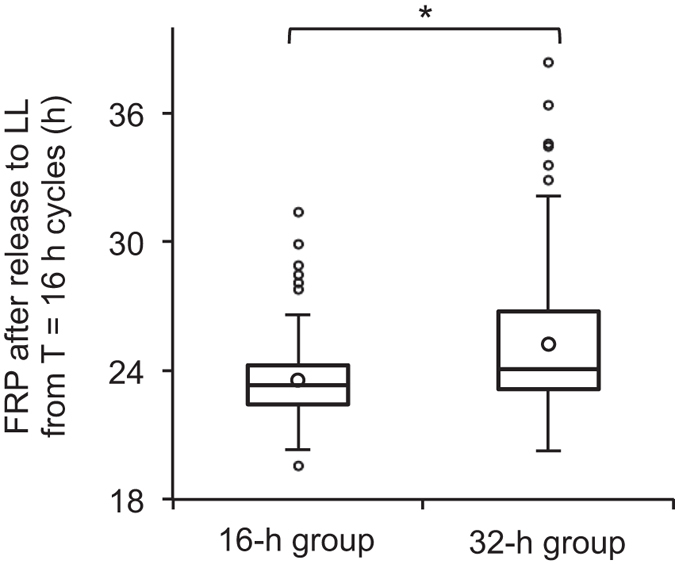
Comparison of fundamental periods under T = 16 h cycles and FRPs under LL. Bioluminescence traces of the *AtCCA1::LUC* reporter were obtained by the single-cell bioluminescence monitoring under T = 16 h cycles, and then were released into LL. See Supplementary Fig. 9 for details of the release experiment. Individual traces with fundamental periods of shorter than 20 h and longer than 25 h were categorised into the 16-h group (*n* = 115) and the 32-h group (*n* = 81), respectively. Boxplots show FRPs of bioluminescence traces with RAE values less than 0.4 for the two groups. Three horizontal lines in each box represent 75%, median and 25% from the top to the bottom. A circle in each box represents the mean value. The bar ends represent the lowest datum still within 1.5× interquartile range of the lower quartile, and the highest datum still within 1.5× interquartile range of the upper quartile. Circles outside of the boxes represent outliers of 1.5× interquartile range. Three experiments were conducted. *p < 0.01, the significant difference between the mean values is based on a two-level nested ANOVA model with two groups (16-h or 32-h group), and subgroups (individual plants in the three experiments) nested within the groups.

The entrainment to T = 16 h cycles was heterogeneous among cells (the 16-h and 32-h groups) of the control samples; the bioluminescence rhythms of *LgELF3-RNAi*-transfected cells were uniformly entrained to this T-cycle (Fig. 3f). The average FRP of the cellular rhythms of the control was longer by ~4 h than that of *LgELF3-RNAi*-transfected cells (Fig. 1c; Table 1). Furthermore, the variation in the FRPs was large, even in the control (σ = 2.3 h, Table 1)^2^. Thus, we determined the correlation between FRPs and the entrainment to T = 16 h cycles in the control samples. We compared fundamental periods of cells under T = 16 h cycles and their FRPs after release to LL (Fig. 4; Supplementary Fig. 9). The average FRP of cells in the 32-h group was 25.3 h, while that of the 16-h group was 23.5 h, which was a statistically significant difference. This indicated that cells in the 16-h group showed a tendency with shorter FRPs. The difference in FRP might be reflected by the phase of the rhythms at the onset of LL. In the release experiments, we set two distinct release points: the light onset of the cycle in which most cells of the 32-h group showed a higher peak (release at 224 h, Supplementary Fig. 9), or the light onset of the cycle containing a lower peak (release at 176 h, Supplementary Fig. 9). The difference in the release point was unlikely to affect the FRPs of the cells in either group (Supplementary Fig. 9). Thus, it was likely that the heterogeneity of the entrainment of cellular circadian rhythms to T = 16 h cycles was associated with the variation in their FRPs. We further checked the first and second peak times after release to LL. The first peaks of the cellular bioluminescence rhythms in the 32-h group came ~5 h after either release point, while the second peak times differed by ~3 h between these release points (Supplementary Fig. 9). Thus, the T = 16 h cycle was unable to completely reset the cellular rhythms in this group to the same phase. Meanwhile, the cellular rhythms in the 16-h group appeared to be completely reset, irrespective of the release points (Supplementary Fig. 9).

## Discussion

In this study, we characterized the entrainment habits of circadian clocks of duckweed at the single cell level. Individual cellular clocks were entrained in an FRP-dependent manner to light/dark cycles with a range of period lengths, and *LgELF3* was involved in the entrainment process by maintaining the proper FRPs of cellular circadian rhythms and by gating the light signal.

**Figure 5:**
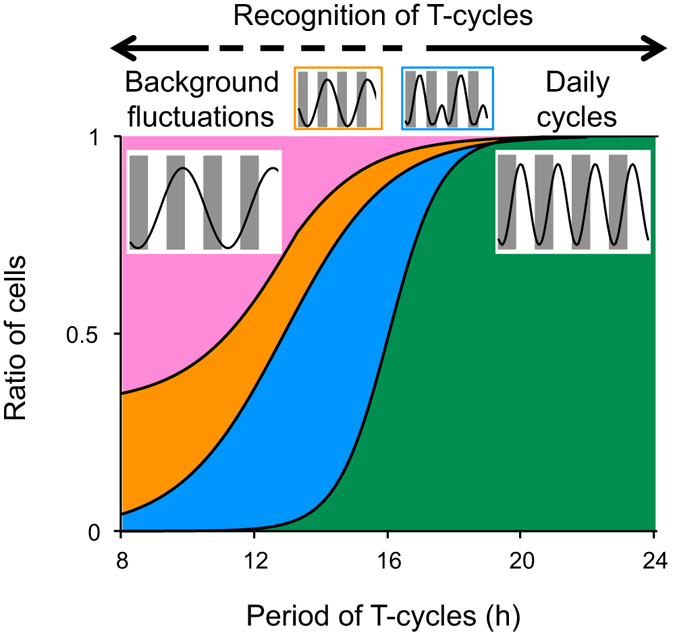
Recognition of T-cycles by a plant composed of cells with heterogeneous circadian properties. Schematic representation of changes in the ratios of cells showing different entrainment habits to environmental T-cycles is shown as a stacked area plot with a hypothetical idea about the recognition of T-cycles by a plant at the top. The four areas represent ratios of cellular rhythms that are entrained (green), incompletely entrained (blue), frequency-demultiplied (orange), and seemingly freerun (pink). Patterns of rhythms in these categories are presented. The plot is based on the data shown in Supplementary Fig. 7.

Changes in the ratios of cellular rhythms of different entrainment habits under various T-cycles are summarized in Fig. 5. It is presumed that the ratios are the basis of the recognition of environmental light/dark cycles by the plant. Although individual cells in the same *L. gibba* tissue showed the circadian rhythms of *AtCCA1::LUC* with a relatively wide range of FRPs (Fig. 1), the heterogeneity of FRPs was almost eliminated under light/dark cycles with periods of 24 h or 20 h^2^. The cellular rhythms were entrained and orderly in the same tissue (Fig 3a–d)^2^. However, fundamental periods of cellular rhythms under T = 16 h cycles were roughly classified into two groups (16 h and 32 h), suggesting that the heterogeneity of entrainment among individual cells was actualized under T = 16 h cycles (Fig. 3f; Supplementary Fig. 5). Since bioluminescence traces in the 32-h group looked to fluctuate every light/dark cycle, it was likely that most cellular clocks were still kept in synchrony with the T = 16 h cycles. This suggests that *L. gibba* recognizes this T-cycle as a day. Cells in both groups were evenly scattered throughout a frond (Supplementary Fig. 8), suggesting that the heterogeneity was dependent not on the positions of the cells. Under LL, FRPs of cellular clocks did not show a dependence on the positions^2^. This seems to coincide with the scattering of the two groups under the T = 16 h cycles. In the release experiment, the average FRP of cells in the 32-h group was longer than that in the 16-h group (Fig. 4). This indicated that the entrainment heterogeneity among cellular clocks at least partly depended on their FRPs. However, cellular clocks with a range of FRPs belonged to both groups, implying that other factors would cause the heterogeneity. Cell-cell interaction for the entrainment would be a possible factor. A phase-attractive interaction between neighbouring cellular clocks was proposed in the duckweed circadian system^2^, and such cell-cell interaction for cellular clocks might contribute to the separation of cells with various FRPs into the two groups under T = 16 h cycles.

In general, circadian clocks can skip a light/dark or temperature cycle(s) if the environmental cycle period is short, for example, about half the length of the FRP; this phenomenon has been described as a hallmark of clock entrainment in many organisms^9, 17, 19–21^. In our experiment, under T = 12 h cycles, the bioluminescence trace of the control sample showed a rhythm with a period length longer than 24 h at the whole plant level (Fig. 2d; Supplementary Fig. 3). Indeed, many cells in the control samples having T = 12 h cycles showed robust rhythms with periods of 24 h or longer, suggesting that the *L. gibba* was unable to recognize this T-cycle as a day. Those cellular rhythms with a period of 24 h showed 1:2 synchronization (frequency demultiplication). This suggests that their phases responded to light/dark transitions to be entrained to the T-cycle, thought the phase transition is latent. Meanwhile a portion of cells showed rhythms apparently responding to every light/dark cycle (Supplementary Fig. 6). Thus, individual cellular clocks are likely to have the ability to maintain their rhythmicity in a plant that is exposed to short light/dark cycles, but the robustness against such light/dark fluctuations varies among cells. Since daily environmental cycles include unpredictable background fluctuations, such as transient gloom from clouds passing the sun, it is important for the circadian clock to be entrained properly to the daily cycles without being perturbed by such short period fluctuations.

Heterogeneity of physiological factors, such as light responsiveness, among cells would also alter the entrainment processes of cellular clocks. The heterogeneity found in FRPs and the light responsiveness among cellular clocks might be due to a difference in clock-related gene expression levels between cells. *ELF3* is an important clock gene in *Arabidopsis*, and this gene is involved in the control of circadian rhythm FRPs^7, 9^. In our experiments using duckweed, the FRPs of cellular circadian rhythms were shortened by the *LgELF3* knockdown and lengthened by its overexpression (Fig. 1; Table 1). Thus, *LgELF3* can be involved in the maintenance of the FRPs of the cellular circadian clock through its expression level. Furthermore, unlike cells of the control sample, *LgELF3-RNAi*-transfected cells were uniformly entrained to T = 16 h cycles (Fig. 3e and f; Supplementary Fig. 5), suggesting that the short period facilitated the entrainment of these cellular clocks to the shorter T-cycle. Notably, when plants were transferred to T = 12 h cycles, the bioluminescence traces of most *LgELF3-RNAi*-transfected cells reached their first peak during the first light period, whereas the control cells did not show obvious peaks during that light period (Time 30–36 h in Fig. 3g). This was reminiscent of the defect in the circadian gating of phototransduction during subjective night in the *Arabidopsis elf3* mutant^12^. Thus, *LgELF3* is likely to be involved in the circadian-gating function of the *L. gibba* cellular clock system. The system appears to work to avoid the perturbations caused by short-period fluctuations in light conditions by maintaining both the FRPs and the light responsiveness of individual cellular clocks in an appropriate range. The circadian system of a plant is likely to permit heterogeneity in the circadian properties among cells, with the condition that cellular clocks can be synchronous under the ~24-h daily cycle without being perturbed by background fluctuations.

Recently the hierarchical circadian structure of the *Arabidopsis* plant has been reported^22, 23^. Circadian clocks of the vasculature and the shoot apex are robust and dominate over clocks in other tissues like mesophylls/leaves. In our experiments using duckweed, bioluminescence rhythms were mainly derived from mesophyll cells^5^. Although their clocks appear to be weakly and locally coupled in the mesophyll^2^, the vasculature/shoot apex may contain strongly-coupled clocks in *L. gibba*. The hierarchical circadian structure in plant would affect the entrainment habits of individual cellular rhythms under environmental light/dark conditions.

## Methods

### Plant materials and growth conditions

The *L. gibba* p8L strain is a pure line (eight generations of selfing) produced from the *L. gibba* G3 strain^24^. Plants were kept on NF medium with 1% sucrose as previously described^2^. Plants were cultured under LL at 25 ± 1 °C with light supplied by fluorescent lamps (FLR40SEX-W/M/36-HG; NEC) at approximately 50 μE·m^−2^·s^−1^.

### Reporter and effector genes

*pUC-AtCCA1::LUC* + (*AtCCA1::LUC*+) was used as the circadian bioluminescent reporter^5^. As effector constructs, *LgELF3-OX*, *LgELF3-RNAi* and *pBI221ΔGUS* were used for *LgELF3H1* overexpression, *LgELF3H1* knockdown and their control, respectively^13^. The *pUC18-Cas9-R1R2* vector was constructed as a derivative of *pDe-Cas9* ^16^ for transient gene expression, and it lacked the right and left borders, as well as the *bar* gene. The oligoDNAs, 5′-attgGAGAGGGAGAGATGAAGATA-3′ and 5′-aaacTATCTTCATCTCTCCCTCTC-3′, were annealed, subcloned into the *pEn-Chimera* vector^16^, and then cloned into the *pUC18-Cas9-R1R2* to make *pUC18-sgLgELF3-Cas9* (sg*LgELF3*). The *pUC18-Cas9* was the control vector and included only the Cas9-expressing region of *pUC18-Cas9-R1R2*.

### Particle bombardment experiment

The bioluminescent reporter gene, with an effector gene, was introduced into plants through particle bombardment. A total of 8 μl of prewashed gold particle suspension (1 μm diameter; Bio-Rad) in 50% glycerol (60 mg·ml^−1^) was mixed with 2 μl of reporter DNA (1 μg μl^−1^), 1 μl effector DNA (1 μg·μl^−1^), 6 μl of spermidine (0.1 M) and 15 μl of CaCl_2_ (2.5 M) in a tube. Preparation of the DNA-coated gold particles and the transfection by a helium gun device (PDS-1000/He; Bio-Rad) were conducted as described previously^2^.

### Bioluminescence monitoring

Bioluminescence monitoring at the whole plant level was performed as described previously^24^. The single-cell bioluminescence imaging was performed as described previously^2^. To illuminate the samples during the single-cell monitoring the optical fibres guided the white light (30 μE·m^−2^·s^−1^) from a light-emitting diode device (PFB-20SW, CCS Inc.).

### Time series analysis

A time series analysis was performed in the statistical environment of R 3.2.2 (http://www.R-project.org/). The FRPs and RAEs of bioluminescence traces were estimated by the fast Fourier transform non-linear least squares method as described previously^2^, using the data in the time ranges described in the figure legends. The peak and the trough times of bioluminescence traces were estimated using R script for peak and trough picking, as described previously^2^. The autocorrelation functions of bioluminescence traces were calculated with the R function *acf* using the data in the time ranges described in the figure legends. A nested ANOVA was carried out using the R function *aov.*

## Electronic supplementary material

Supplementary Information
