# Supplementary material for: Synchrony of plant cellular circadian clocks with heterogeneous properties under light/dark cycles
Source: Sci Rep. 2017 Mar 22;7:317. doi: 10.1038/s41598-017-00454-8 (PMC5428515; doi:10.1038/s41598-017-00454-8)
Supplement: Supplementary file 1 — Supplementary Information [file 41598_2017_454_MOESM1_ESM.pdf]

Supplementary Information

Title: Synchrony of plant cellular circadian clocks with heterogeneous properties under light/dark cycles

Authors/Affiliation:

Masaaki Okada, Tomoaki Muranaka, Shogo Ito, and Tokitaka Oyama*

Department of Botany, Graduate School of Science, Kyoto University, Kitashirakawa-oiwake-cho, Sakyo-ku, Kyoto 606-8502, Japan

Corresponding Author:

Tokitaka Oyama, PhD

Department of Botany, Graduate School of Science, Kyoto University,

Kitashirakawa-oiwake-cho, Sakyo-ku, Kyoto 606-8502, Japan

Tel: 81-75-753-4135

E-mail address: oyama@cosmos.bot.kyoto-u.ac.jp

Supplementary Figures 1-9

Supplementary Fig. 1

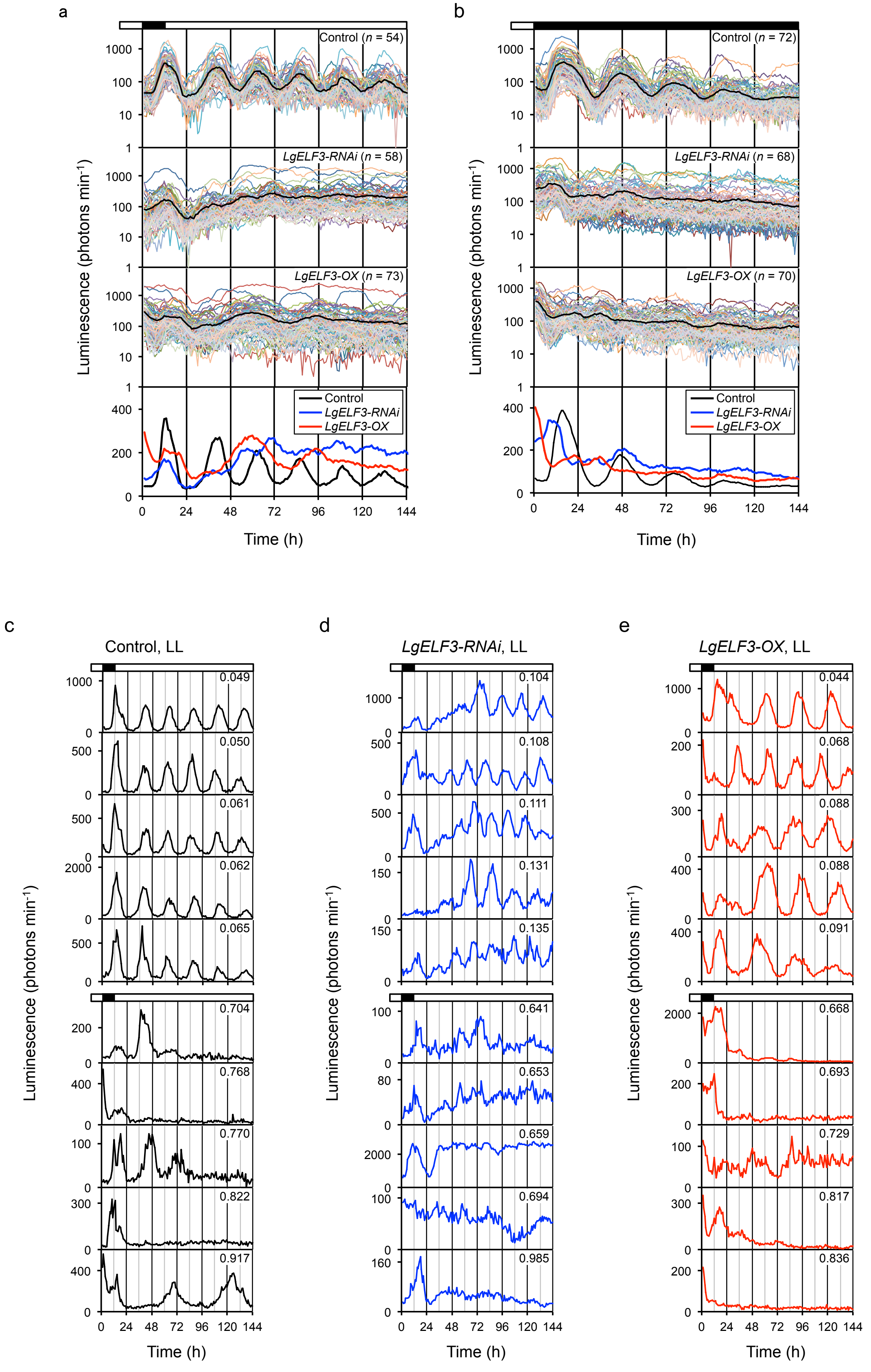

Supplementary Fig. 1 (continued)

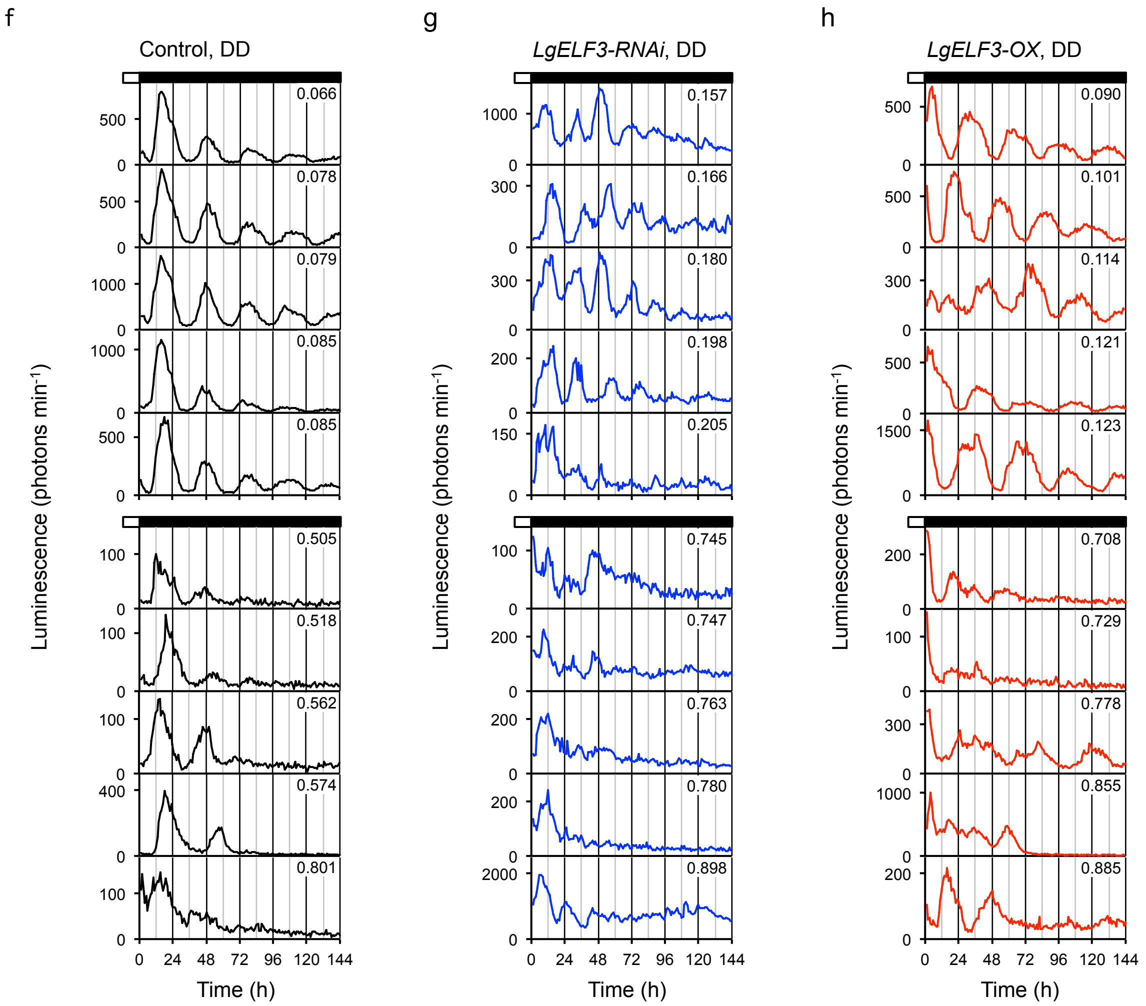

Supplementary Fig. 1. Bioluminescence traces of the *AtCCA1::LUC* reporter in individual cells under constant conditions.

(a, b) Effects of co-transfection of an effector, the knockdown *LgELF3-RNAi* (*second from the top*) or the overexpressing *LgELF3-OX* (*third from the top*), compared with the control vector (*top*) on cellular circadian rhythms of the *AtCCA1::LUC* reporter. Bioluminescence traces of the *AtCCA1::LUC* reporter obtained by the single-cell bioluminescence monitoring under LL (a) or DD (b). The luminescence intensity of every measured cell on a frond are plotted. Three experiments were conducted for each condition. A black line in each graph represents the average bioluminescence of the total traces. A comparison of these average bioluminescence traces is shown in the bottom graph. The number of samples is indicated in each graph. (c-h) Examples of bioluminescence traces in individual cells: Control (c, f), *LgELF3-RNAi* (d, g) and *LgELF3-OX* (e, h) under LL (c-e) or DD (f-h). Those cellular bioluminescence traces with the five lowest- and the five highest RAE values under each condition in the three experiments are shown in ascending order. The RAE value is indicated in each graph. RAEs and FRPs of bioluminescence traces were estimated by the fast Fourier transform non-linear least squares method using the data in the 48 h to 144 h (LL) or 24 h to 120 h (DD) time ranges. For all of the measured cells, these values are shown in Figs 1a and 1b. White and black bars above graphs represent light and darkness, respectively.

Supplementary Fig. 2

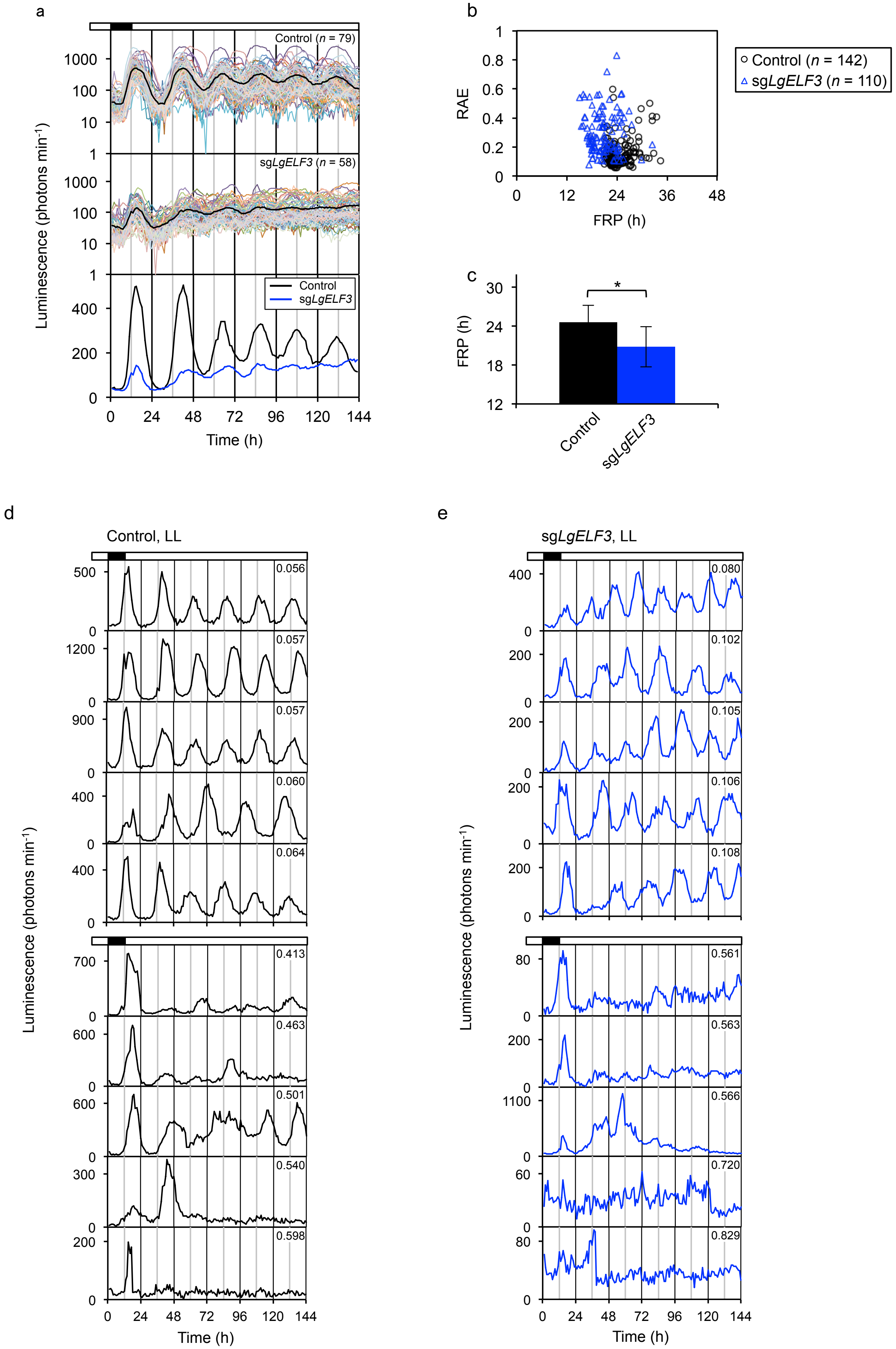

Supplementary Fig. 2. Effects of mutations in the *LgELF3* locus induced by a CRISPR/Cas9 system on the *AtCCA1::LUC* rhythms of individual cells under LL**.**

(a) Bioluminescence traces of the *AtCCA1::LUC* reporter obtained by the single-cell bioluminescence monitoring under LL. The control vector for a CRISPR/Cas9 system (*pUC18-Cas9*, *top*) or the sg*LgELF3* construct (*middle*) was introduced into *L. gibba* plants together with the reporter. A black line in each graph represents the average bioluminescence of the total traces. A comparison of these average bioluminescence traces is shown in the bottom graph. The number of samples is indicated in each graph. (b) FRPs and RAEs of individual cellular rhythms obtained from the two independent experiments are plotted. (c) Comparison of FRPs shown in (b) for the cellular rhythms with RAE value less than 0.4. Data are expressed as mean ± SD values. *$p < 0.01$, the significant difference between the mean values is based on a two-level nested ANOVA model with two groups (effector constructs: control, sg*LgELF3*), and subgroups (individual plants in the two experiments) nested within the groups. (d, e) Examples of bioluminescence traces of individual cells: Control (d) and sg*LgELF3* (e). The cellular bioluminescence traces with the five lowest- and the five highest RAE values in the two experiments are shown in ascending order. The RAE value is indicated in each graph. White and black bars above graphs represent light and darkness, respectively.

Supplementary Fig. 3

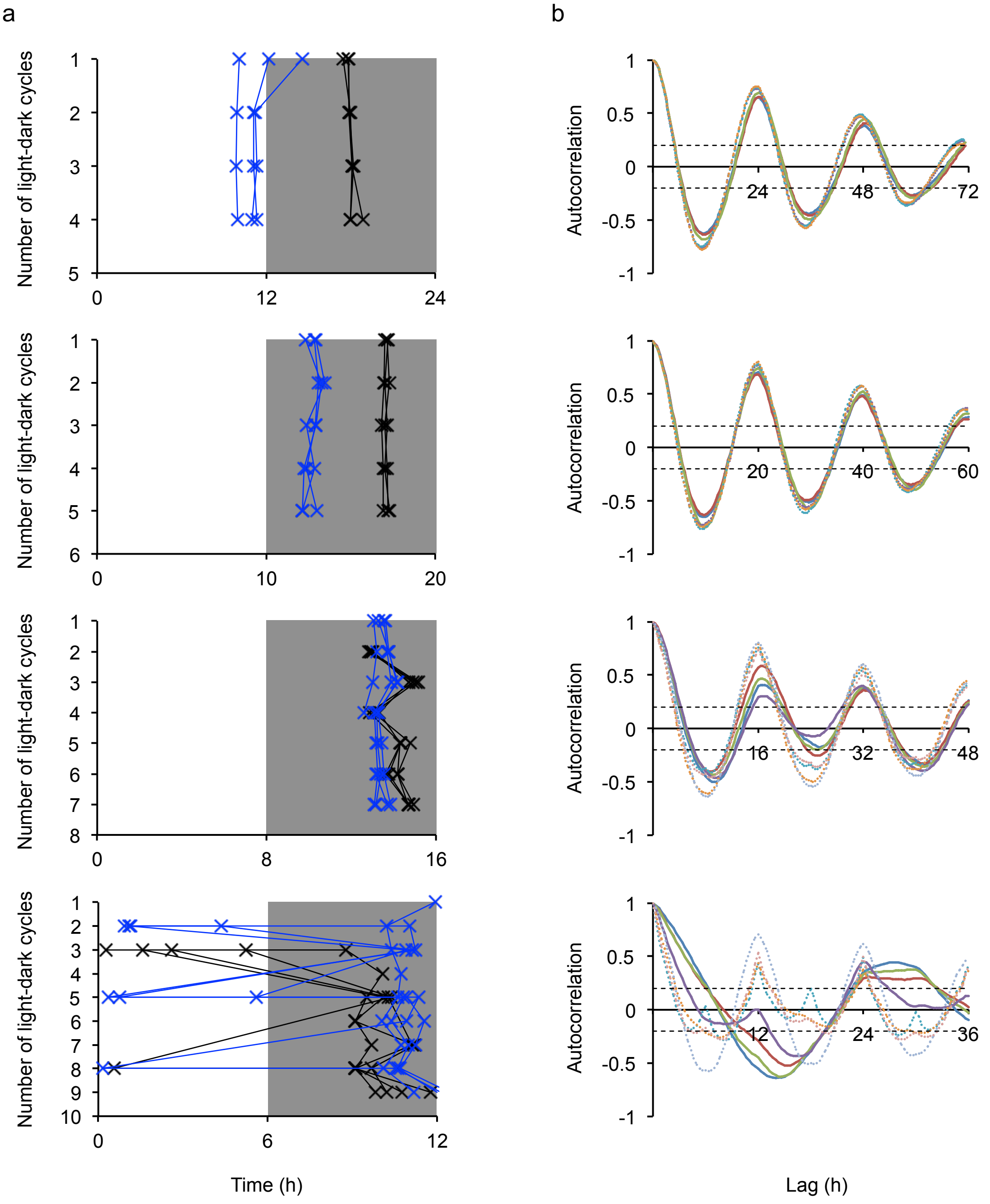

Supplementary Fig. 3. Trough phases and autocorrelation functions of *AtCCA1::LUC* rhythms under various T-cycle conditions at the whole plant level.

(a) Estimated trough times of bioluminescence traces in Fig. 2 were plotted for T = 24 h, 20 h, 16 h and 12 h (top to bottom). Black and blue lines represent trough times of bioluminescence traces of the control and the *LgELF3* knockdown, respectively. Trough times were estimated and plotted for each bioluminescence trace from three or four replicate dishes at every light-dark cycle under each condition. The times of the 1st light-dark cycles correspond to the following times: 36–60 h (Fig. 2a) for T = 24 h, 34–54 h (Fig. 2b) for T = 20 h, 32–48 h (Fig. 2c) for T = 16 h and 30–42 h (Fig. 2d) for T = 12 h. Shaded boxes indicate the duration of the dark periods in the light-dark cycles. (b) The autocorrelation function of each bioluminescence trace in Fig. 2 is shown [T = 24 h, 20 h, 16 h and 12 h (top to bottom)]. The autocorrelation function was calculated using the data in the 48 h to 144 h time range. Solid lines and dotted lines represent autocorrelation functions of samples of the control and the *LgELF3* knockdown, respectively. The horizontal dashed black lines in each graph represent the borders of the 95% confidence intervals of the autocorrelation function.

Supplementary Fig. 4

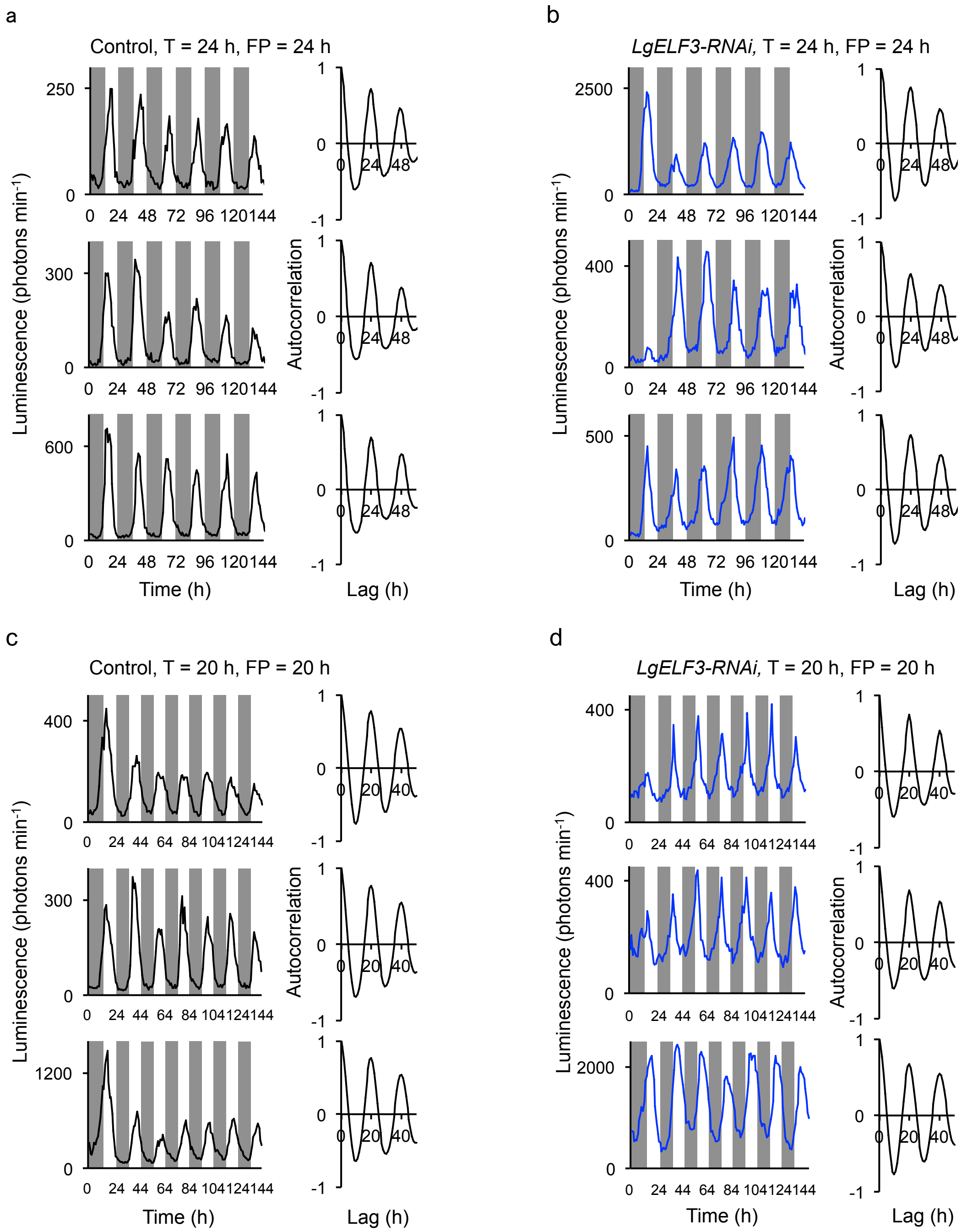

Supplementary Fig. 4. Examples of *AtCCA1::LUC* rhythms of individual cells under T = 24 h and 20 h cycles.

Representative bioluminescence traces of individual cells in Figs 3a and c are shown on the graphs in the left-side panels using the colors black (control) and blue (*LgELF3* knockdown), and their corresponding autocorrelation functions are shown in the graphs in the right-side panels. Bioluminescence traces with the three highest autocorrelation peaks of the control samples (a, c) or the *LgELF3* knockdown (b, d) under T = 24 h (a, b), T = 20 h (c, d). Information about the sample (Control or *LgELF3-RNAi*), the period of T-cycles (T) and the fundamental period (FP) is indicated. Shaded boxes indicate the duration of darkness. The autocorrelation function of each trace was calculated using the data in the 48 h to 144 h time range.

Supplementary Fig. 5

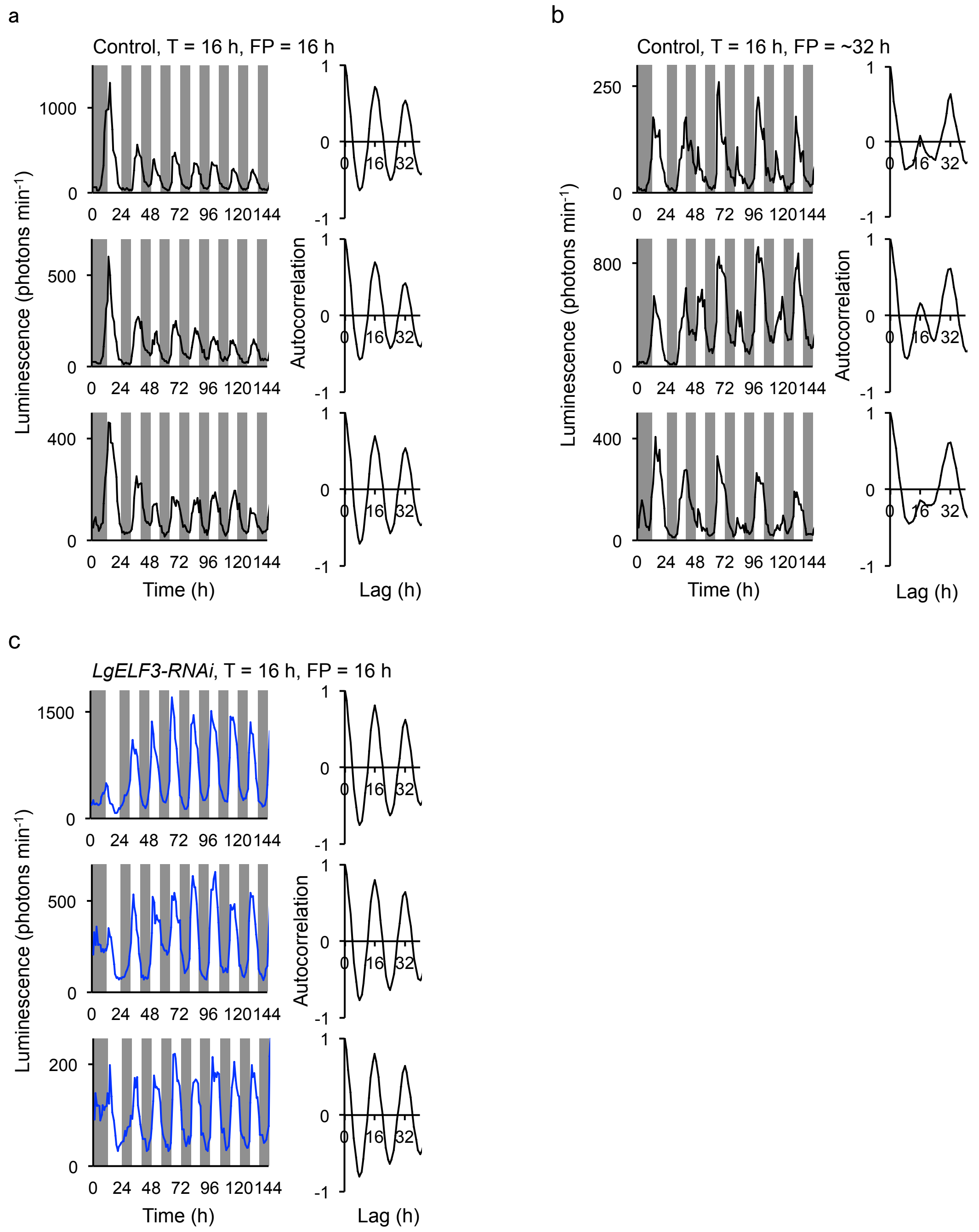

Supplementary Fig. 5. Examples of *AtCCA1::LUC* rhythms of individual cells under T = 16 h cycles.

Representative bioluminescence traces of individual cells in Fig. 3e are shown on the graphs in the left-side panels using the colors black (control) and blue (*LgELF3* knockdown), and their corresponding autocorrelation functions are shown in the graphs in the right-side panels. Bioluminescence traces with the three highest autocorrelation peaks of the control samples (a, b) or the *LgELF3* knockdown (c) under T = 16 h on the following stipulation. The fundamental periods were the periods of 16 h (a, c) or ~32 h (b). Information about the sample (Control or *LgELF3-RNAi*), the period of T-cycles (T) and the fundamental period (FP) is indicated. Shaded boxes indicate the duration of darkness. The autocorrelation function of each trace was calculated using the data in the 48 h to 144 h time range.

Supplementary Fig. 6

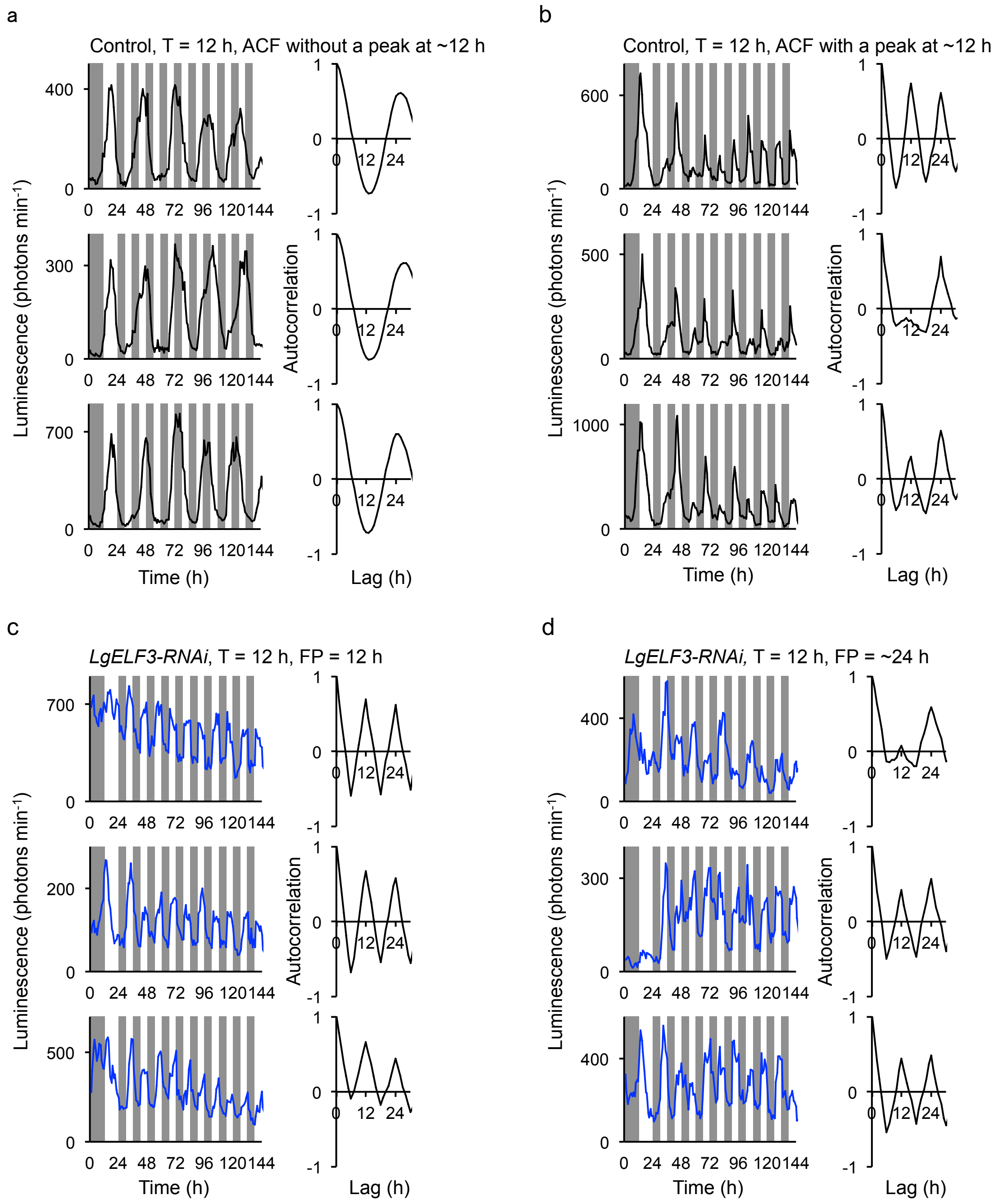

Supplementary Fig. 6. Examples of *AtCCA1::LUC* rhythms of individual cells under T = 12 h cycles.

Representative bioluminescence traces of individual cells in Fig. 3g are shown on the graphs in the left-side panels using the colors black (control) and blue (*LgELF3* knockdown), and their corresponding autocorrelation functions are shown in the graphs in the right-side panels. Bioluminescence traces with the three highest autocorrelation peaks of the control samples (a, b) or the *LgELF3* knockdown (c, d) under T = 12 h on the following stipulations. The autocorrelation function (ACF) lacked a peak at ~12 h time lag (a). The ACF peaked both at ~12 h- and ~24 h time lags (b, c, d). The fundamental periods (FP) were the periods of 12 h (c) or ~24 h (d). Information about these stipulations together with the sample (Control or *LgELF3-RNAi*) and the period of T-cycles (T) is indicated. Shaded boxes indicate the duration of darkness. The autocorrelation function of each trace was calculated using the data in the 48 h to 144 h time range.

Supplementary Fig. 7

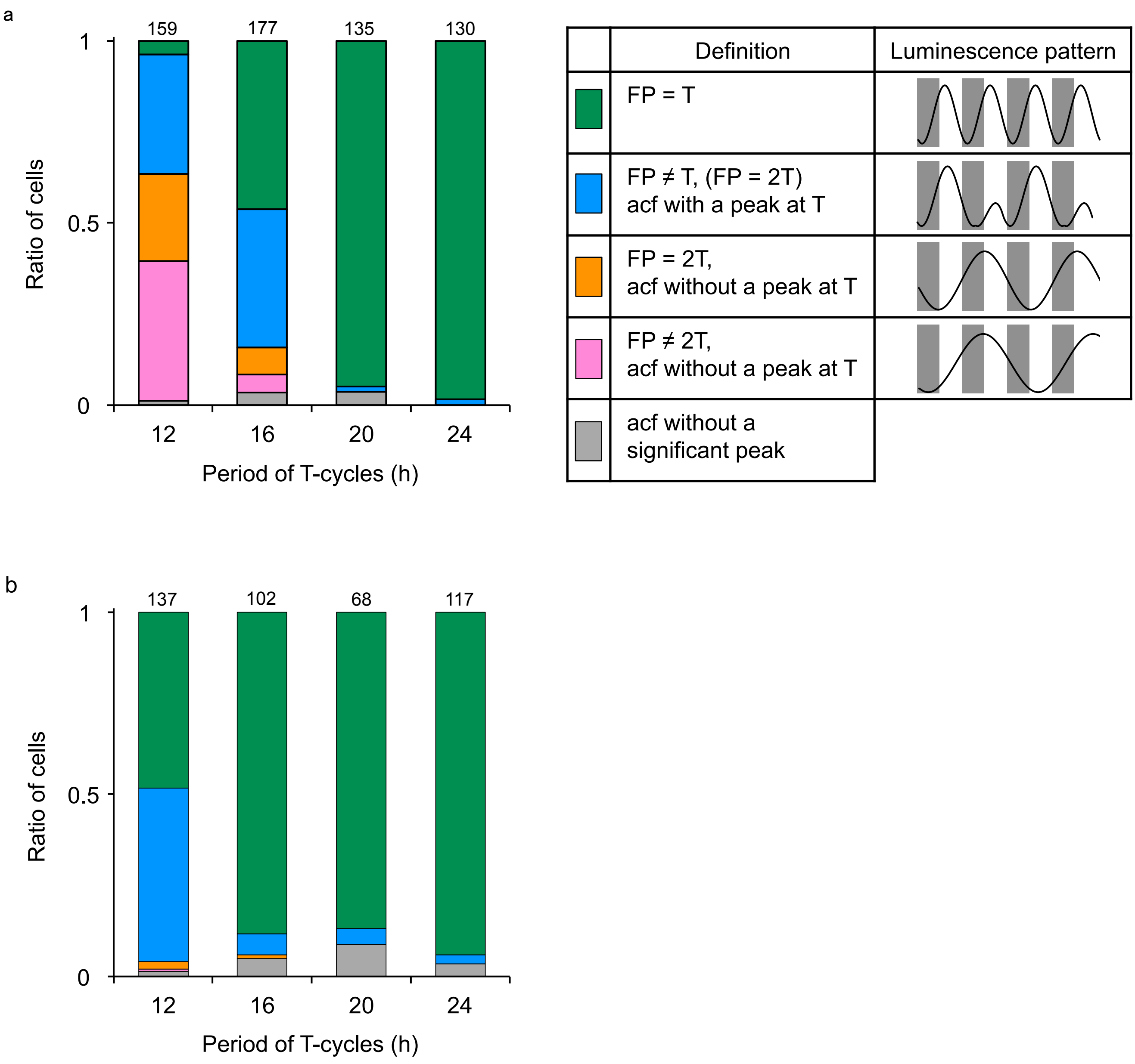

Supplementary Fig. 7. Comparison of entrainment habits of cellular rhythms under various T-cycles.

Ratios of cells showing different entrainment habits in each T-cycle condition are plotted in stacked bar graphs for the control samples (a) or the *LgELF3* knockdown (b). The entrainment habit of each cellular rhythm in a T-cycle condition is classified into one of the five categories that are based on the fundamental period (FP) and the autocorrelation function (ACF) at the time lag of T as explained in the graph legend. Definitions of the categories and schematic luminescence patterns are shown in the graph legend. The number of total evaluated cells in each condition is indicated above the bar.

Supplementary Fig. 8

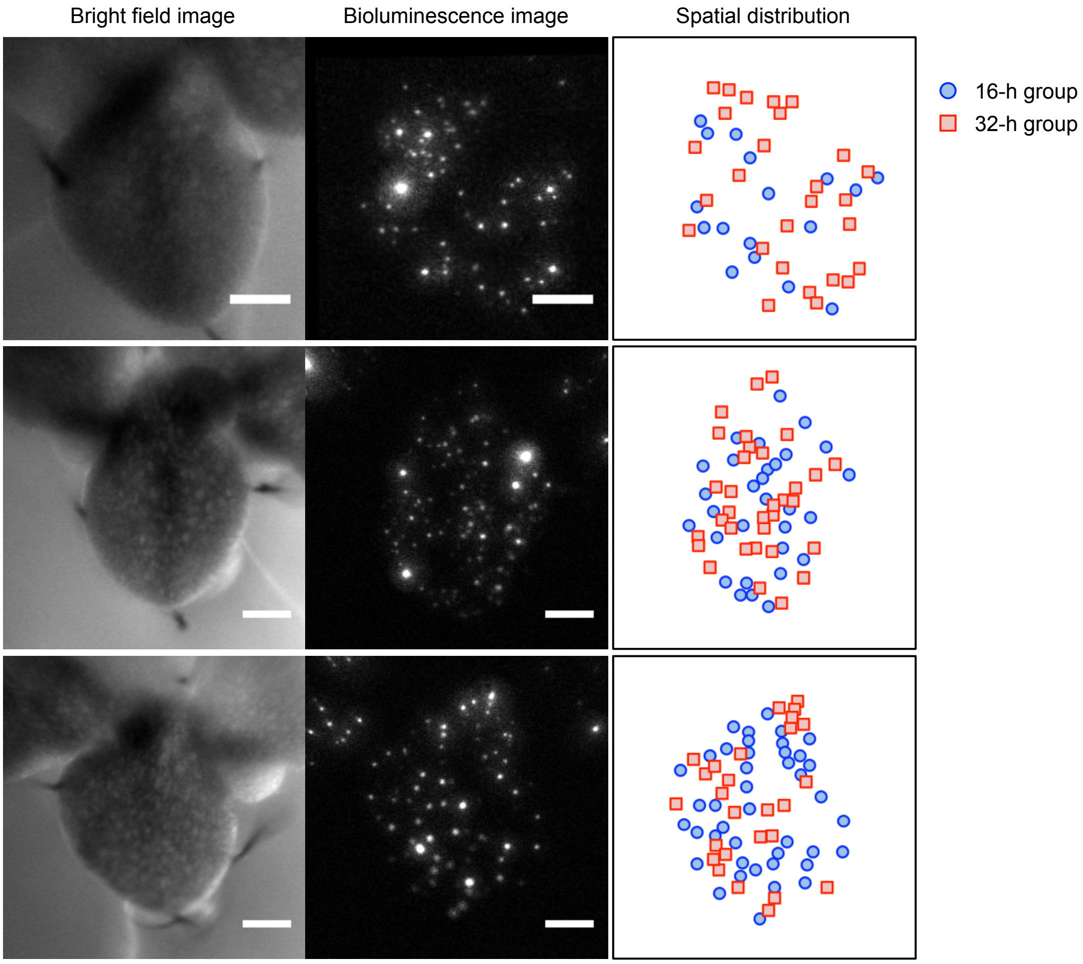

Supplementary Fig. 8. Spatial distribution of cells with fundamental periods of ~16 h or ~32 h under T = 16 h cycles.

Three fronds transfected with the *AtCCA1::LUC* reporter together with the control vector were analyzed for cellular bioluminescence rhythms under T = 16 cycles [shown in Figs 3e, f (control)]. Bright field images (left), bioluminescence images (middle), and the spatial distributions of cells (right) in the 16-h group (blue circles) or the 32-h group (red squares). Scale bars = 1 mm.

Supplementary Fig. 9

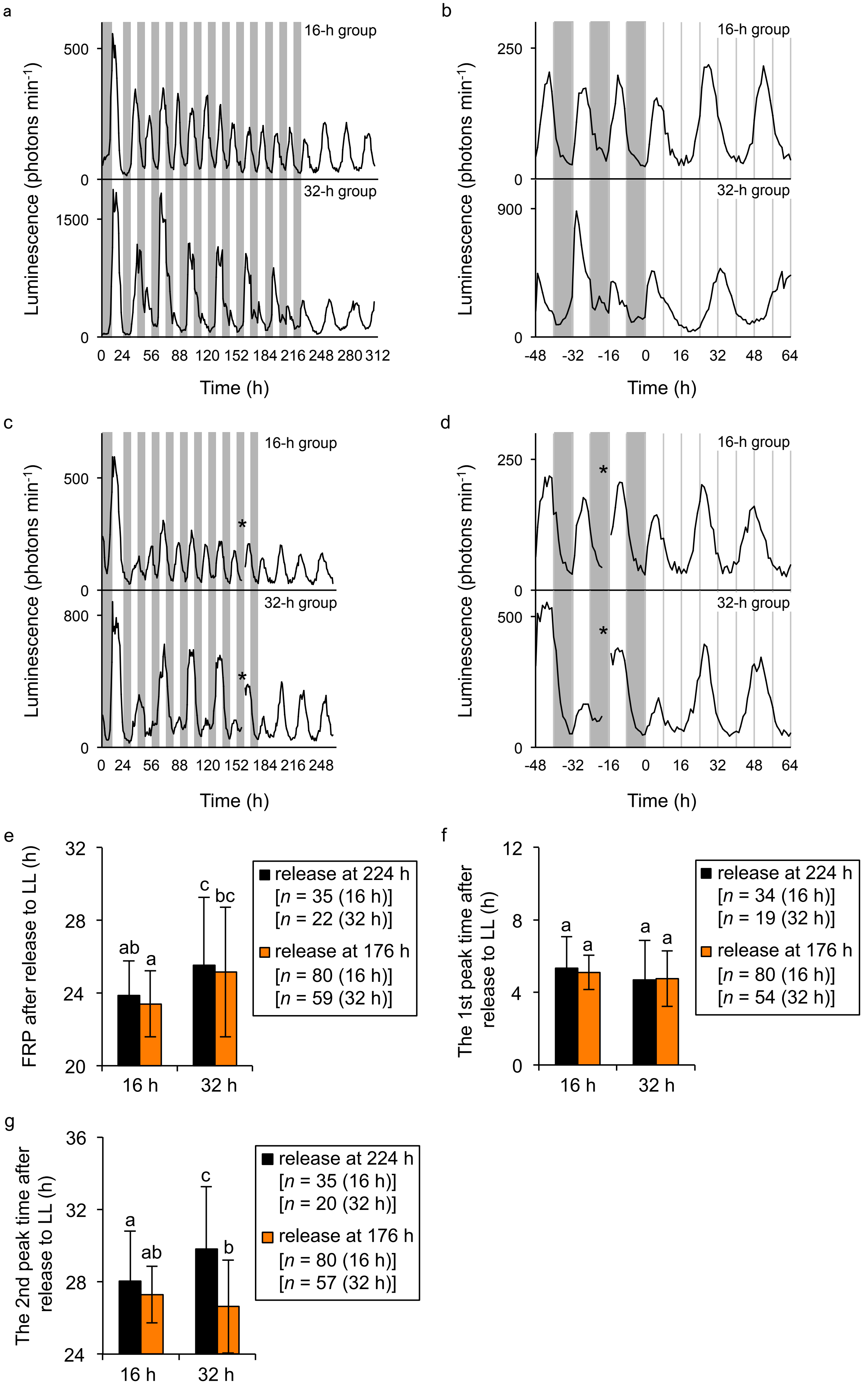

Supplementary Fig. 9. Release experiments from T = 16 h cycles to LL.

(a-d) Examples of the *AtCCA1::LUC* bioluminescence traces from individual cells having T = 16 h cycles and then under LL. Traces in the 16-h group of the fundamental period (upper panel) and the 32-h group (lower panel) under T = 16 h cycles are shown. Light conditions were changed from T = 16 h cycles to LL at 224 h (a) or 176 h (c). Traces from near the release time points of (a) and (c) are shown in (b) and (d), respectively. Time 0 h represents the release points (b, d). Shaded boxes indicate the duration of darkness. *Data at three time points are missing due to a mechanical error. The autocorrelation function of each bioluminescence trace was calculated using the data from 48 h to 144 h. (e, f, g) Quantification of circadian properties of bioluminescence traces shown in (a) and (c) after release to LL. Means for FRP (e), the first peak times (f), and the second peak times (g) in the release experiments were calculated for each group of fundamental periods, and those with release points at 224 h (a) and 176 h (c) are represented as black and orange bars, respectively. Every two mean values were compared using a two-level nested ANOVA model with four groups (16-h or 32-h group released at 224 h or 176 h), and subgroups (individual plants in three experiments) nested within the groups. Means with the same letters are not significantly different. FRPs of bioluminescence traces were estimated by the fast Fourier transform non-linear least squares method using the data from 236 h to 308 h (a) or 188 h to 260 h (c). Bioluminescence traces with RAE values less than 0.4 in LL were used for the analysis.
